# Supplementary material for: AffyMAPSDetector: a software tool to characterize Affymetrix GeneChip™ expression arrays with respect to SNPs
Source: BMC Bioinformatics. 2007 Jul 30;8:276. doi: 10.1186/1471-2105-8-276 (PMC1959249; doi:10.1186/1471-2105-8-276)
Supplement: Additional file 13 — AffyMAPSDetector v1 source code. AffyMAPSDetector source code. [file 1471-2105-8-276-S13.zip › AffyMAPSDetector_v1_src/doc/affychip/snpdetector/model/ProbeSet.html]

ProbeSet


|  |  |  |  |
| --- | --- | --- | --- |
| **Overview** \| **Package** \| **Class** \| **Use** \| **Tree** \| **Deprecated** \| **Index** \| **Help** | | |  |
| **PREV CLASS**   **NEXT CLASS** | **FRAMES**    **NO FRAMES**     **All Classes** |  |  |
| SUMMARY: NESTED \| FIELD \| CONSTR \| METHOD | DETAIL: FIELD \| CONSTR \| METHOD |  |  |


---


## affychip.snpdetector.model Class ProbeSet

```
java.lang.Object
  affychip.snpdetector.model.ProbeSet
```

**All Implemented Interfaces:**: java.lang.Comparable

---

public class **ProbeSet** extends java.lang.Object implements java.lang.Comparable

Title: Probe SNP Detector

Description: Affymetrix Gene-Probe SNP Detection

All the probes corresponding to a particular gene on a Affy chip.

Copyright: Copyright (c) 2004

Company: George Mason University

**Version:**
:   1.1

**Author:**
:   Sunita Kumari

---

**Constructor Summary**

`ProbeSet(java.lang.String probesetId)`


|  |  |
| --- | --- |
| **Method Summary** | |
| `void` | `addProbe(Probe aProbe)` |
| `int` | `compareTo(java.lang.Object o)` |
| `Probe` | `getProbeFor(java.lang.String x, java.lang.String y)` |
| `java.util.Collection` | `probeSNPs(java.lang.String snps)` |
| `java.util.Collection` | `probeSNPs(java.lang.String[] snpList)` |
| `void` | `setGeneInfo(GeneInfo geneInfo)` |
| `java.lang.String` | `toString()` |

**Methods inherited from class java.lang.Object**

`clone, equals, finalize, getClass, hashCode, notify, notifyAll, wait, wait, wait`

**Constructor Detail**

### ProbeSet

```
public ProbeSet(java.lang.String probesetId)
```


**Method Detail**

### setGeneInfo

```
public void setGeneInfo(GeneInfo geneInfo)
```

---


### addProbe

```
public void addProbe(Probe aProbe)
```

---


### probeSNPs

```
public java.util.Collection probeSNPs(java.lang.String snps)
```

---


### probeSNPs

```
public java.util.Collection probeSNPs(java.lang.String[] snpList)
```

---


### getProbeFor

```
public Probe getProbeFor(java.lang.String x,
                         java.lang.String y)
```

---


### toString

```
public java.lang.String toString()
```

---


### compareTo

```
public int compareTo(java.lang.Object o)
```

:   **Specified by:**: `compareTo` in interface `java.lang.Comparable`


---


|  |  |  |  |
| --- | --- | --- | --- |
| **Overview** \| **Package** \| **Class** \| **Use** \| **Tree** \| **Deprecated** \| **Index** \| **Help** | | |  |
| **PREV CLASS**   **NEXT CLASS** | **FRAMES**    **NO FRAMES**     **All Classes** |  |  |
| SUMMARY: NESTED \| FIELD \| CONSTR \| METHOD | DETAIL: FIELD \| CONSTR \| METHOD |  |  |


---
